# Outcomes of second-line combination antiretroviral therapy for HIV-infected patients: a cohort study from Rio de Janeiro, Brazil

**DOI:** 10.1186/s12879-014-0699-5

**Published:** 2014-12-19

**Authors:** Sandra W Cardoso, Paula M Luz, Luciane Velasque, Thiago S Torres, Isabel C Tavares, Sayonara R Ribeiro, Ronaldo I Moreira, Valdilea G Veloso, Richard D Moore, Beatriz Grinsztejn

**Affiliations:** Instituto Nacional de Infectologia Evandro Chagas, Fundação Oswaldo Cruz, Rio de Janeiro, Brazil; Departamento de Matemática e Estatística, Universidade Federal do Estado do Rio de Janeiro, Rio de Janeiro, Brazil; Johns Hopkins University, Baltimore, Maryland USA

**Keywords:** cART, Second-line, Cox proportional hazards regression, HIV/AIDS, Cohort study, Brazil

## Abstract

**Background:**

World-wide, the notable expansion of HIV/AIDS treatment programs in resource-limited settings has lead to an increasing number of patients in need of second-line cART. To adequately address and prepare for this scenario, critical assessments of the outcomes of second-line cART are particularly relevant in settings where monitoring strategies may be inadequate. We evaluated virologic outcomes of second-line combination antiretroviral therapy (cART) among HIV-infected individuals from Brazil.

**Methods:**

This study was conducted at the Instituto Nacional de Infectologia Evandro Chagas, Fundação Oswaldo Cruz, at Rio de Janeiro, Brazio. For this study we included all patients who started first-line and second-line cART between 2000 and 2013. Second-line cART required a switch in the anchor drug of first-line cART. We evaluated time from second-line start to virologic failure and factors associated with increased risk of failure using multivariable Cox proportional hazards regression models.

**Results:**

Among the 1,311 patients who started first-line cART a total of 386 patients (29.5%) initiated second-line cART, out of which 35.0% and 60.6% switched from their first-line to their second-line cART when their HIV RNA was undetectable and after documented virologic failure, respectively. At second line cART initiation, median age was 38 years [interquartile range (IQR): 31-45years]. Median CD4 count was significantly different for patients starting second-line cART undetectable [412 cells/mm^3^ (IQR: 240-617)] compared to those starting second-line cART after documented virologic failure [230 cells/mm^3^ (IQR: 118-322.5)] (p < 0.01). Median time from second-line cART initiation to failure was also significantly different for patients starting second-line cART undetectable compared to those who with documented virologic failure (log-rank test p < 0.01). Multivariable Cox models showed that younger age, lower education, and HIV RNA level were independently associated with an increased hazard of second-line failure among those with documented virologic failure at start of second-line cART.

**Conclusions:**

We have shown that in a middle-income country with universal access to cART, having a detectable HIV RNA at the start of second-line cART as well as younger age and lower education negatively impact second-line outcomes. Our findings could guide HIV treatment efforts as to which strategies would help maximize the durability of these regimens.

**Electronic supplementary material:**

The online version of this article (doi:10.1186/s12879-014-0699-5) contains supplementary material, which is available to authorized users.

## Background

Globally, studies addressing second-line combination antiretroviral (cART) virologic, immunologic and clinical outcomes have become increasingly common in recent years [1]–[5]. Resource-limited settings have expanded their HIV/AIDS treatment programs leading to an increasing number of patients in need of second-line cART. Currently, boosted protease inhibitors (PI)-containing regimens are the recommended option after first-line cART failure for patients treated in the public sector in most resource-limited settings [6]. Given the first-line cART usually available in resource-limited settings, concerns over the development of resistance to second-line cART have been raised, particularly in the absence of (or in the presence of insufficient) viral load monitoring [7],[8]. Indeed, the emergence of drug resistance has been a major threat to the sustained effectiveness of cART in resource-rich settings [9] and the continuation of a failing regimen may be associated with more complex mutation patterns as observed in several studies [10]–[12]. A critical assessment of the outcomes of second-line cART in resource-limited settings is timely as treatment programs are relatively new or maturing and monitoring strategies are still inadequate [13],[14].

Second-line cART accounts for less than 5% of total antiretroviral treatments in resource-limited settings [15]. In Latin America and the Caribbean region, the percentage of individuals receiving second-line cART is higher than that reported in other resource-limited settings (27% of the patients, ranging from 4 to 43%, are receiving second-line regimens compared with 0.05% in other regions of world) [16]. This may in part be due to specific characteristics of the Americas region, such as the age of national programs, with many patients starting cART before 2000, as well as with an access to a broader options of drugs. In 1996, Brazil was the first middle-income country to implement a universal access cART while also providing immunologic and virologic monitoring and resistance testing after first-line failure. Currently, over 300,000 patients are on cART, using first-, second-, third-line and salvage regimens, and roughly 30,000 patients initiate treatment yearly. Nevertheless, no data is available on second-line treatment outcomes within the routine care provided through the public health system. An understanding of factors associated with second-line cART outcomes in our setting is critical to guide the evolution of the Brazilian HIV Treatment Program, as well as to maximize the durability of these regimens, preventing disease progression and reducing mortality. Accordingly, this study describes second-line cART outcomes in a large urban cohort from Rio de Janeiro, Brazil, including time to failure as well as factors associated with treatment failure.

## Methods

### Description of the clinical cohort

This study was conducted at the Instituto Nacional de Infectologia Evandro Chagas, Fundação Oswaldo Cruz (INI/FIOCRUZ, formerly known as Evandro Chagas Clinical Research Institute), where care has been provided to HIV/AIDS patients since 1986. INI is one of the largest infectious diseases research centers in Brazil, where over five thousand HIV infected patients have been treated since the beginning of the AIDS epidemic. An observational, longitudinal, clinical database is maintained on patients receiving primary and specialized HIV care at the clinic. Details and results of the HIV/AIDS Clinical Cohort can be found elsewhere [17]. INI’s Institutional Review Board reviewed and approved the study and patients provided written informed consent.

### Study population and definitions

For this study we included all patients who started first-line cART between January 01, 2000 and June 30, 2010. Follow-up information included data up to December 30, 2013. Included patients had to be at least 18 years old at cohort enrollment and have followed first-line cART by a second-line cART regimen. First- and second-line cART regimens were defined as two or more nucleoside reverse transcriptase inhibitors (NRTI) plus an anchor drug [i.e., a nonnucleoside reverse transcriptase inhibitor (NNRTI), or a protease inhibitor (PI)]. Patients were treated according to the Brazilian HIV Treatment Guidelines that recommend as a preferred first-line cART a regimen based on NNRTIs though PI-based regimens can also be prescribed. In contrast, second-line cART, according to the guideline, should be PI-based though NNRTI-based regimens can also alternatively be prescribed [18].

Second-line cART was defined for individuals who either failed first-line cART (HIV RNA viral load measurement > 400 copies/mL after 5 months of initiating first-line cART) and subsequently switched their regimen class and/or drug, depending on the class of the first-line cART as detailed below, or for patients who had not failed their first-line cART but switched their regimen class and/or drug, depending on the class of the first-line cART as detailed below. Patients who started a first-line NNRTI-based regimen were assumed to have switched to second-line cART if a PI-based regimen was started. Alternatively, patients who started a first-line PI-based regimen were assumed to have switched to second-line cART if either a NNRTI-based regimen was started or there was a switch of the PI drug used. Modifications to the NRTIs backbone, that is, in any or all NRTIs alone, were not considered as switches from first to second-line cART. Also, the addition of ritonavir to a PI-based first-line regimen was not considered as a switch from first to second line cART.

Start of follow-up for this analysis is defined by the start date of the second-line cART. The definition of virologic failure for second-line cART was conditional on the HIV RNA level at the start of the second-line cART regimen. For patients with an HIV RNA of >400 copies/ml at the start of second-line cART, virologic failure was defined as an HIV RNA level of >400 copies/ml after 5 months of start of second-line cART. For patients with an HIV RNA of ≤ 400 copies/ml at the start of second-line cART, virologic failure was defined as an HIV RNA > 400 copies/ml after 2 months of start of second-line cART. Two scenarios were defined as virologic failure while on second line cART: a single HIV RNA > 400 copies/mL followed by a regimen class modification as well as having two consecutive HIV RNAs > 400 copies/mL regardless if a regimen class modification had occurred or not. A single HIV RNA > 400 copies/mL followed by a subsequent HIV RNA of < 400 copies/mL with no regimen class modification was considered a blip. The median time between the two HIV RNA measurements was 4 months (mean of 6 months, interquartile range 2-7 months). Standard of care adherence counseling was provided by the attending physician once virologic failure was detected, as per local guidelines. Deaths from AIDS-related causes were defined as virologic failures while deaths from non-AIDS-related causes were defined as censored observations. Censoring was also applied in two situations. First, patients who did not fail assuming the definitions above were censored at the date of the last viral load plus six months. Second, patients who did not have a an HIV RNA result or any documented switch in regimen class were censored after the grace periods described above (patients with an HIV RNA of > 400 copies/ml at the start of second-line cART were censored at 5 months and patients with an HIV RNA of < 400 copies/ml at the start of second-line cART were censored at 2 months). The limit of detection of viral load assays used throughout the study period varied from < 400 to < 50 copies/ml. For consistency, we have used the <400 copies/ml threshold for the entire study period.

### Genotype testing

For this study, we included genotype testing results available in patient’s medical chart. Genotype testing was performed at the Laboratory of AIDS and Molecular Immunology, at the Oswaldo Cruz Institute, which is part of RENAGENO (the Brazilian National Network for genotyping, a network of laboratories implemented in 2001 by the Ministry of Health). The genotyping assays used were ViroSeq® and TrueGene®. Both tests employ DNA sequencing methodology and are approved and validated; most tests were performed with TRUGENE Kit®.

### Statistical analysis

Basic bivariate analysis included performing the Pearson’s Chi-square test for categorical variables and the Mann-Whitney rank sum test for continuous variables. Kaplan-Meier plots and the log-rank test were used to analyze the time to predefined outcomes. Cox proportional hazards regression models were fitted to estimate relative hazards and 95% confidence intervals of time to second-line cART virologic failure. To verify the proportionality assumption in Cox’s model, we tested the correlation between survival time and Shoenfeld’s standardized residuals. R software version 3.0.3 (www.r-project.org) was used for all statistical analyses.

## Results

Among the 1,311 patients who started first-line cART, a total of 386 patients (29.5%) initiated second-line cART. Most patients used a first-line NNRTI-based regimen (n = 243; 63%) while 143 (37%) used a PI-based regimen, 51% (74/143) of these a non-boosted PI. First-line effectiveness for our cohort of patients has been previously described [19].

Out of 386 patients who started second-line cART, 135 (35.0%) patients switched from their first-line to second-line cART when their HIV RNA was undetectable, and 234 patients (60.6%) initiated second-line cART after documented virologic failure (Table 1). Seventeen patients (4.4%) did not have an HIV RNA result available before second-line cART initiation. Reasons for switching first-line cART were available for 382 patients. The most frequent reason for switching first-line cART were toxicities (121/382 [31.7%]), 41% and 26% among the undetectable and detectable groups, respectively. Other reasons for switching first-line cART were therapeutic failure (including virologic, immunologic or clinical failure, 115/382 [30.1%]), physician’s decision (including drug-drug interaction, actual or desired pregnancy, or to improve adherence or for dose convenience, 100/382 [26.2%]), and patient’s decision (46/382, 12.0%).

**Table 1:** **Demographic and clinical characteristics at second-line cART initiation stratified by HIV RNA level, 2000-2013**

			HIV RNA level		
		Overall	Undetectable	Detectable	p-value
N (%)*		369	135	234	
**Age**					
	Median (IQR)	38.3 (31.8-45.2)	38.7 (30.4-45.4)	37.9 (31.9-45.2)	0.93
	≤30	78 (21.1)	33 (24.4)	45 (19.2)	0.27
	30-39	127 (34.4)	40 (29.6)	87 (37.2)	
	≥40	164 (44.4)	62 (45.9)	102 (43.6)	
**Race**					0.05
	Non white	185 (50.1)	58 (43.0)	127 (54.3)	
	White	184 (49.9)	77 (57.0)	107 (45.7)	
**Gender/HIV exposure**					0.28
	Heterosexual men	110 (28.5)	36 (26.7)	70 (30.7)	
	Women	127 (33.0)	50 (37.0)	71 (31.1)	
	MSM	97 (25.0)	38 (28.1)	57 (25.0)	
	IDU	9 (2.5)	4 (3.0)	5 (2.2)	
	Other	37 (10.0)	7 (5.2)	25 (11.0)	
**Education**					0.11
	≤8 years	219 (59.5)	72 (53.7)	147 (62.8)	
	>8 years	149 (41.5)	62 (46.3)	87 (37.2)	
**Years since first HIV+ test**					0.80
	Median (IQR)	3.3 (1.8-5.8)	3.1 (1.7-5.8)	3.4 (1.9-5.7)	
**CD4 cell count, cells/mm3**					<0.01
	Median (IQR)	260 (145, 425)	412 (239, 617)	230 (118, 322)	
	≤200	110 (34.3)	19 (15.6)	91 (45.7)	
	201-350	96 (29.9)	31 (25.4)	65 (32.7)	
	>350	115 (35.8)	72 (59)	43 (21.6)	
HIV RNA level*					
	Log10 copies/ml, median (IQR)	N/A	N/A	4.4 (3.8-5.0)	-
	≤400 copies/ml	135 (36.6)	135 (100.0)	N/A	-
	401-10,000 copies/ml	83 (22.5)	N/A	83 (35.5)	
	10,001-100,000 copies/ml	97 (26.3)	N/A	97 (41.5)	
	>100,000 copies/ml	54 (14.6)	N/A	54 (23.0)	
**Change in anchor drug from 1** ^**st**^ **to 2** ^**nd**^ **line**					<0.01
	NNRTI to PI-r	204 (55.3)	50 (37)	154 (65.8)	
	NNRTI to PI	27 (7.3)	13 (9.6)	14 (6)	
	PI/PI-r to NNRTI	73 (19.8)	42 (31.1)	31 (13.2)	
	PI/PI-r to PI/PI-r	65 (17.6)	30 (22.2)	35 (15)	
**Calendar year**					0.97
	2000-2006	154 (41.7)	57 (42.2)	97 (41.5)	
	2007-2013	215 (58.3)	78 (57.8)	137 (58.5)	
**Days from 1** ^**st**^ **line failure to 2** ^**nd**^ **line initiation**					-
	Median (IQR)	N/A	N/A	173 (0.0-606)	

*17 patients did not have an HIV RNA result prior to starting second-line cART.

*Abbreviations: HIV* human immunodeficiency virus, *MSM* men who have sex with men, *IDU* injection drug user, *ART* antiretroviral therapy

Gender and reported mode of HIV exposure were categorized jointly into women, heterosexual men, MSM, IDU (men and women) and other reported modes of HIV exposure (men and women). Individuals reporting both IDU and other modes of HIV exposure were categorized into IDU.

Education was self-reported and based on the number of years of formal education.

CD4 cell count was defined as the CD4 cell count closest to the date of start the second line cART up to 30 days after.

HIV RNA was defined as the HIV RNA measurement closest to the date of start second line cART up to 7 days after.

Days from failure of first-line cART to start of second-line cART was assumed to be zero if patients started second-line cART without virologic failure.

At second line cART initiation, median age was 38 years [interquartile range (IQR): 31-45years], 59% were male and half were non-white (Table 1). The majority (59%) of patients had less than 8 years of formal education and the median time since first HIV-positive test was 3.3 years. Overall, median CD4 count at second-line cART initiation was 260 cells/mm^3^ (IQR: 145-425 cell/mm3). The median CD4 count was significantly different for patients starting second-line cART undetectable [412 cells/mm^3^ (IQR: 240-617)] compared to those starting second-line cART after documented virologic failure [230 cells/mm^3^ (IQR: 118-322.5)] (p < 0.01, Table 1). The median HIV RNA at the time of second-line for those with virologic failure was 4.4 log_10_ copies/mL (IQR: 3.8-5.0 log_10_ copies/mL).

Most anchor drug switches were either a modification from NNRTI- or PI-based regimens to a boosted PI-based regimen (73%) and to a much smaller extent from a PI/PI-r to an NNRTI (19%). Overall, 296 patients (82%) used a PI-based second-line cART out of which the majority (269/296, 91%) used a ritonavir-boosted PI (PI-r). Lopinavir/r was used by 46% (135/296) and ATV/r by 37% (111/296). The most frequently used NRTI backbones were: AZT/3TC (197/369, 53%) and 3TC/TDF or FTC/TDF (114/369, 31%). For patients initiating second-line cART with an undetectable HIV RNA, the most frequent switches were from NNRTI-based to a boosted PI-based cART (37%) followed by PI/PI-r to NNRTI-based (31%) and PI/PI-r to PI/PI-r (22%). For patients who initiated second-line cART with a detectable HIV RNA, the most frequent modification was from NNRTI to a boosted PI-based (65%) cART (Table 1).

**Figure 1:**
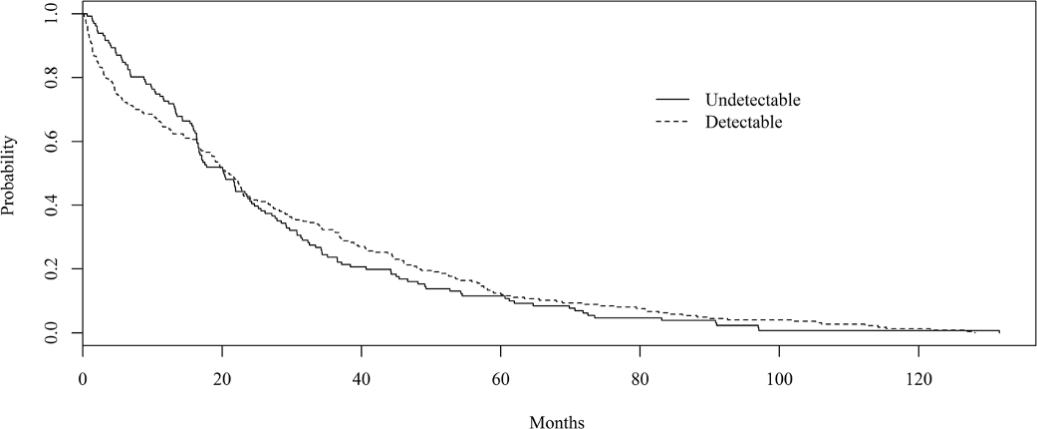
**Time from first-line cART initiation to second-line cART initiation stratified by HIV RNA level at time of second-line cART initiation, IPEC cohort, 2000-2013.** Log-rank test indicated that curves were not significantly different (p = 0.58).

Overall, the median time from first-line cART to second-line cART was 20.1 months (95% CI 17.1-22.6). For patients who initiated second-line cART with an undetectable HIV RNA, the median time was 20.3 months (95% 16.7-24.2) and for those who initiated cART with a detectable HIV RNA, the median time was 20.8 months (18.4-23.1). The Kaplan-Meier plot of the time from first-line cART to second-line cART is given in Figure 1; log-rank test indicated that the curves were not significantly different (p = 0.58).

Among the 386 patients who started second-line cART (median follow-up time of 1.9 years), 199 patients developed virologic failure, 174 were censored, and 13 patients died during follow-up due to AIDS-related causes (5 and 6 deaths in the undetectable and detectable groups). The overall incidence of second-line failure was 12/1000 person-years (95% CI 10-14/1000 person-years). The incidence of second-line failure was 5.7/1000 person-years (95% CI 4.2-7.7/1000 person-years) and 19/1000 person-years (95% CI 16-23/1000 person-years) among those who started second line cART with an undetectable and a detectable HIV RNA (incidence rate ratio for detectable compared to undetectable of 3.4, p < 0.0001). The overall probability of failure at 12, 24 and 36 months was 26%, 41% and 48% respectively. For the undetectable group, 12-, 24- and 36-months probability of failure was 15%, 23% and 30% while for the detectable group, 12-, 24- and 36-months probability of failure was 34%, 55% and 62%.

**Figure 2:**
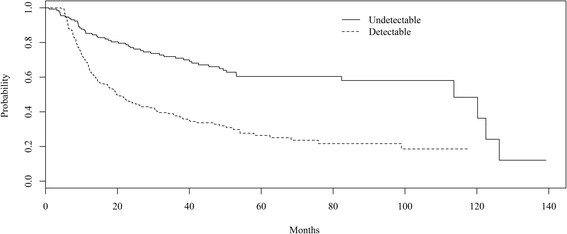
**Time from second-line cART initiation to virologic failure stratified by HIV RNA level at time of second-line cART initiation, IPEC cohort, 2000-2013.** Log-rank test indicated that curves were significantly different (p < 0.01).

**Figure 3:**
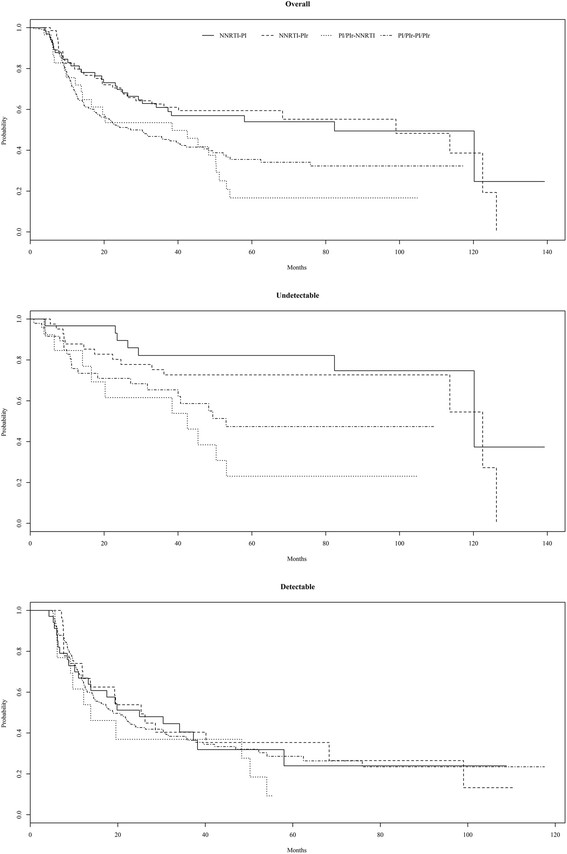
**Time from second-line cART initiation to virologic failure stratified by anchor drug received for all patients (top, log-rank test p = 0.002), the sub-group of patients with an undetectable viral load (middle, log-rank test p < 0.002), and the sub-group of patients with detectable viral load (bottom, log-rank test p = 0.70), IPEC cohort, 2000-2013.**

Overall, the median time from second-line cART to virologic failure was 40.0 months (95% CI 30.4, 52.2). The median time from second-line cART initiation to failure was significantly different between the two groups (log-rank test p < 0.01, Figure 2). For patients who started second-line cART undetectable, the median time was 113.6 months (lower bound of the 95% CI 82.4 months, upper bound could not be calculated) and for those who started second-line cART detectable the median time was 19.8 months (95% CI:15.7-28.6 months). Overall, the median time from second-line cART initiation to failure stratified by anchor drug was significantly different (log-rank test p = 0.002, Figure 3). The median time from second-line cART initiation to failure stratified by anchor drug was also significantly different for the undetectable group (log-rank test p < 0.002). In contrast, for the detectable group, the median time from second-line cART initiation to failure stratified by anchor drug was not significantly different (log-rank test p = 0.70). No differences were observed in the median time from second-line cART to AIDS related deaths (25.2 months [95% CI:10.1-50.8 months] and 23.4 months [95% CI:12.1-40.2] for the detectable and undetectable groups, respectively [log rank test p = 0.22]).

### Factors associated with second-line cART failure

The hazard ratios (HR) for second-line failure stratified by HIV RNA level at second-line cART initiation (detectable and undetectable) are shown in Table 2. For patients who initiated second-line cART with an undetectable HIV RNA, age, second-line anchor drug and first-line duration were independently associated with the hazard of second-line failure (Table 2). A protective effect was observed for older age, with a lower HR for those individuals older than 40 years when compared to those younger than 30 years at second-line initiation (HR 0.44 95% CI 0.22-0.90), as well as for switching either from PI/PI-r to NNRTI (HR 0.40 95% CI 0.18-0.85) and PI/PI-r to PI-r (HR 0.39 95% CI 0.15-0.96) when compared to switching from a NNRTI to a PI-r based regimen. The longer the patient remained on first-line cART, the lower was the hazard for second-line failure, with a 3% decrease in the hazard of failure for each additional month on first line cART. Toxicity as the reason for switching first-line cART was not associated with an increased incidence of second-line cART failure.

**Table 2:** **Adjusted hazard ratios and 95% confidence intervals as estimated by Cox proportional hazards regression for second-line cART failure stratified by HIV RNA level at time of second-line cART initiation, 2000-2013**

		Undetectable	Detectable
**Age**			
	<30 years	Ref.	Ref.
	30-39 years	0.75 (0.34-1.70)	0.54 (0.35-0.83)
	≥40 years	0.44 (0.22-0.90)	0.58 (0.38-0.89)
**Education**			
	>8 years		Ref.
	≤8 years		1.44 (1.01-2.03)
**HIV RNA level, copies/ml**			
	401-10,000		Ref.
	10,001-100,000		1.42 (0.98-2.06)
	>100,000		1.09 (0.70-1.70)
**Second-line cART anchor agent**			
	NNRTI-PI/r	Ref.	
	NNRTI-PI	1.19 (0.53-2.70)	
	PI/PI/r-NNRTI	0.40 (0.18-0.85)	
	PI/PI/r-PI/PI-r	0.39 (0.15-0.96)	
**Time from first-line to second-line initiation**			
	Per 1 month increase	0.97 (0.95-0.99)	

For patients who initiated second-line cART with a detectable HIV RNA, age and level of education were independently associated with the hazard of second-line failure. Again, a protective pattern was found for older age when compared to younger individuals and for higher education when compared to those with less years of education (Table 2). Whilst not significant, a higher hazard of failure was observed for patients initiating second-line with an HIV RNA level between 10,001-100,000 copies/ml, when compared to those with HIV RNA levels <10,000 copies/ml (p = 0.06). Time from first-line cART failure to second-line cART initiation was not associated with the incidence of second-line cART failure.

### Resistance testing

HIV genotyping results at first-line cART failure were available for 39% (91/234) of the patients who started second-line cART with a detectable viral load. A genotyping result yielding wild type virus was found in 7.7% (7/91) of patients. Overall, M184V was the most prevalent mutation (59/91, 65%). K65R was identified in 38% of the patients using TDF (8/21) all of which were using an NNRTI based cART regimen. At least one primary PI mutation was found for 75% (6/8) and 55% (5/9) of patients using PI and PI-r cART based regimens. Among those patients using a NNRTI based regimen, the most prevalent NNRTI mutation was K103N (43/74, 58%).

Resistance testing results were available for 23% (43/187) of the patients at second-line cART virologic failure. Overall, 32.5% (14/43) of the genotyping results yielded no resistance mutations (wild type virus), and this was higher among those patients using a boosted PI cART regimen (12/30, 40%). More than 3 TAMS were identified in 11.6% (5/43). At least one primary PI mutation was identified in 75% (3/4) of patients using an unboosted PI cART based regimen (D30N, I50L and 46 V), and 10% (3/30) among those using a PI-r cART regimen (47 V, 82A and 90 M).

## Discussion

Our results show that more than half of our study population (60%) started second-line cART after a documented virologic failure, and that the outcomes from start of second-line cART were significantly different for patients who started second-line cART with an undetectable compared to a detectable HIV RNA. The median time from second-line cART to failure was significantly higher for those who started with an undetectable HIV RNA compared to those with a detectable HIV RNA. In addition, the incidence of second-line failure was 3.4 times higher for those with a detectable viral load and the probabilities of failure at 12, 24 and 36 months were consistently higher for this group. Taken together, these results clearly point to differences in outcomes as a function of an individual’s HIV RNA at start of second-line cART which is in agreement with the findings from a multicenter study with a similar design [20].

Given that an undetectable HIV RNA can be taken as surrogate marker for treatment response and possibly good adherence and no viral resistance, it was expected that patients starting second-line cART undetectable would show improved outcomes. Indeed, non-adherence to therapy has been shown to be one of the strongest predictors of cART failure [21],[22]. In this group, the median time from second-line cART to failure was 113 months, two-fold higher than the 50 months median time found in a multicenter study from the United States [20]. Differences in socio-demographic characteristics, adherence pattern and regimen tolerability may explain the better outcomes shown in our study. Also, in our cohort, only 2% were IDUs, while up to 24.2% were IDUs in the US cohort [20]. Moreover, our study participants initiated cART on or after the year 2000 when friendlier regimens were increasingly available.

As for socio-demographic factors impacting second-line cART outcomes, our results suggest that older age and higher education were associated with a decreased hazard of failure. For this sample population, age older than 30 years at start of second-line cART significantly decreased the hazard of failure among patients with detectable and undetectable HIV RNA at second-line cART start. In a recent analysis of first-line cART conducted by our group, both factors were also found to impact treatment effectiveness [19] and a higher proportion of viral suppression was detected for older/elderly patients [23]. In agreement, a protective effect of older age was also observed in other cohorts both in resource-rich and resource-limited settings [24],[25]. Older age and higher education are likely correlated with a better understanding of the importance and value of cART and, consequently, better treatment adherence [26]. These results are worrisome given the current epidemiological scenario of the Brazilian AIDS epidemic which shows an increase of AIDS cases among the younger and less educated [18]. A better understanding of the beliefs and barriers to cART adherence as well as interventions tailored to these specific populations to improve cART outcomes are urgently needed.

Our results also show that anchor drug switches from first-line to second-line cART can impact the hazard of second-line cART among patients who had an undetectable HIV RNA at the start of second-line cART. We found that patients switching from an NNRTI-based regimen to an unboosted PI-based regimen had increased hazard of failure of second-line cART when compared to those switching to a boosted-PI-based regimen. In contrast, patients were protected from failure if switches were from unboosted or boosted PI-based regimens to either NNRTI-based regimens or a boosted PI-based regimen. These results echo current knowledge since unboosted PI regimens are no longer an option in contemporary treatment guidelines [6],[18]. Our results show that the hazard of second-line cART failure was lower the longer the patient stayed on first-line cART. These findings can be interpreted as being due to a good treatment adherence which was first evidenced during first-line cART and possibly extends to second-line. In contrast, for the sub-group of patients with a detectable HIV RNA, the HIV RNA level at start of second-line cART led to an increased hazard of failure with borderline significance. Indeed, HIV RNA >100,000 copies/mL is well known to be associated with higher risk of first-line cART virologic failure [27]–[29] and second-line cART failure [30],[31]. We speculate that the higher risk of failure among patients with intermediate HIV RNA level at second-line cART initiation could suggest that this group had more irregular treatment adherence and, consequently, more viral resistance.

Another finding from our study is that the median time from first-line to second-line cART was not significantly different among those who started second-line cART undetectable compared to those who started with a detectable HIV RNA, suggesting that concurrent reasons to switch first-line cART occurred overtime which included toxicities in addition to virologic failure. In fact, a higher proportion of patients who switched to second-line cART while having HIV RNA undetectable did so due to toxicities (41% and 26% among the undetectable and detectable groups, respectively). A somewhat surprising finding was that the median time from second-line cART to failure was significantly longer than that of first-line cART (40 months for second-line compared to 20 months for first-line). This may result from a high proportion of first-line cART switches due to toxicities. In previous analyses we have shown that toxicities were the driving force for first-line cART modifications and/or interruptions which were highly incident in our cohort especially during the first year of cART [32].

Although HIV genotyping tests are available in Brazil through the Public Health System since 2001, only 39% of the patients had a resistance test performed after first-line virologic failure and before starting second-line cART. Also, a small number of patients (23%) who developed second-line cART virologic failure had a resistance testing performed at second-line failure. Several issues such as insufficient laboratory infrastructure, long intervals to receive the genotyping results, technical limitations related to the viral load threshold for viral amplification (initially 5,000 copies/mL, only recently changed to 1,000 copies/mL), among others, may explain the small number of tests performed. Of note, although our numbers are very small, it is important to highlight the number of genotyping results yielding wild type virus among patients using a PI-r cART regimen at second-line virologic failure. Although boosted PI cART regimens have a higher genetic barrier against resistance [33],[34], it is well known that poorer adherence may happen more frequently with this drug class, as a consequence of the worse tolerability profile, especially with lopinavir/r, the most frequently used boosted-PI in our cohort.

Our study has strengths and limitations. A major strength of our study is its large time span, covering 13 years of cART, in a cohort of HIV-infected patients from a middle-income country that provides universal access to treatment. In this scenario we were able to study outcomes of second-line cART with detailed socio-demographic, laboratory and treatment data. To the best of our knowledge, this is the first study of second-line cART outcomes in Brazil and, in the international literature, possibly one of the few studies that longitudinally evaluated outcomes of second-line cART. Lack of adherence data is a major limitation of our study. Despite this pitfall, given the availability of HIV RNA measurements, we were capable of using an undetectable HIV RNA as a proxy for treatment adherence which allowed us to discuss important findings with respect to second-line cART. That said, specific treatment adherence measures are needed to study its impact as well as to allow for adherence interventions to be carried out. Current WHO guidelines recommend that patients presenting with failure should be subject to adherence support interventions, after which a second viral load test should be performed prior to deciding on a regimen switch [6]. In this study, we were not able to consistently use two consecutive HIV RNA measurements as a definition of virologic failure. Instead, we used the definition that was feasible and this could have led to some misclassification of patients as failures when in fact this would not have been confirmed. In addition, beyond study definitions, the reality of having only one measurement available implies that physicians are also making decisions to switch regimens based on only one measurement, meaning that patients might be unnecessarily being subject to treatment modifications. Studies have shown that individuals on first-line cART virologic failure experienced re-suppression without switching [35]. To explore these speculations additional studies are needed.

## Conclusions

Achieving sustained viral suppression is the objective of any cART irrespective if it is the first-line or subsequent treatment regimens. The need for lifelong antiretroviral therapy for HIV infection argues for the use of subsequent regimens with the most favorable efficacy and safety profile, ideally including drug classes without super-imposable resistance patterns. We have shown that in a middle-income country with universal access to cART, having a detectable HIV RNA at the start of second-line cART negatively impacts second-line outcomes and that factors such as younger age and lower education can also negatively impact second-line cART outcomes. Although third-line cARTs such as darunavir, etravirine, raltegravir and maraviroc are available in Brazil, the costs associated with these drugs are significantly higher, and treatment regimens frequently include a larger number of pills, adding further complexity to treatment management. As such, tailored interventions to the specific populations here described are critical to guarantee the benefits of second-line cART.

## Authors’ original submitted files for images

Below are the links to the authors’ original submitted files for images. Authors’ original file for figure 1 Authors’ original file for figure 2 Authors’ original file for figure 3

## References

[1] Fox MP, Ive P, Long L, Maskew M, Sanne I (2010). High rates of survival, immune reconstitution, and virologic suppression on second-line antiretroviral therapy in South Africa. J Acquir Immune Defic Syndr.

[2] Kumarasamy N, Venkatesh KK, Devaleenal B, Poongulali S, Yepthomi T, Solomon S, Flanigan TP, Mayer KH (2011). Safety, Tolerability, and Efficacy of Second-Line Generic Protease Inhibitor Containing HAART after First-Line Failure among South Indian HIV-Infected Patients. J Int Assoc Physicians AIDS Care (Chic).

[3] Pujades-Rodriguez M, Balkan S, Arnould L, Brinkhof MA, Calmy A, MSF AWGo (2010). Treatment failure and mortality factors in patients receiving second-line HIV therapy in resource-limited countries. JAMA.

[4] Siripassorn K, Manosuthi W, Chottanapund S, Pakdee A, Sabaitae S, Prasithsirikul W, Tunthanathip P, Ruxrungtham K, Bamrasnaradura Study T (2010). Effectiveness of boosted protease inhibitor-based regimens in HIV type 1-infected patients who experienced virological failure with NNRTI-based antiretroviral therapy in a resource-limited setting. AIDS Res Hum Retroviruses.

[5] Win MM, Maek ANW, Phonrat B, Kiertiburanakul S, Sungkanuparph S (2011). Virologic and Immunologic Outcomes of the Second-Line Regimens of Antiretroviral Therapy Among HIV-Infected Patients in Thailand. J Int Assoc Physicians AIDS Care (Chic).

[6] Organization WH: Consolidated guidelines on the use of antiretroviral drugs for treating and preventing HIV infection. Recommendations for a public health approach. http://www.who.int/hiv/pub/guidelines/arv2013/en/; 2013., 24716260

[7] Gallant JE (2007). Drug resistance after failure of initial antiretroviral therapy in resource-limited countries. Clin Infect Dis.

[8] Sungkanuparph S, Manosuthi W, Kiertiburanakul S, Piyavong B, Chumpathat N, Chantratita W (2007). Options for a second-line antiretroviral regimen for HIV type 1-infected patients whose initial regimen of a fixed-dose combination of stavudine, lamivudine, and nevirapine fails. Clin Infect Dis.

[9] Napravnik S, Keys JR, Quinlivan EB, Wohl DA, Mikeal OV, Eron JJ (2007). Triple-class antiretroviral drug resistance: risk and predictors among HIV-1-infected patients. AIDS.

[10] Hosseinipour MC, van Oosterhout JJ, Weigel R, Phiri S, Kamwendo D, Parkin N, Fiscus SA, Nelson JA, Eron JJ, Kumwenda J (2009). The public health approach to identify antiretroviral therapy failure: high-level nucleoside reverse transcriptase inhibitor resistance among Malawians failing first-line antiretroviral therapy. AIDS.

[11] Wallis CL, Mellors JW, Venter WD, Sanne I, Stevens W (2010). Varied patterns of HIV-1 drug resistance on failing first-line antiretroviral therapy in South Africa. J Acquir Immune Defic Syndr.

[12] Orrell C, Walensky RP, Losina E, Pitt J, Freedberg KA, Wood R (2009). HIV type-1 clade C resistance genotypes in treatment-naive patients and after first virological failure in a large community antiretroviral therapy programme. Antivir Ther.

[13] Boyd MA, Cooper DA (2007). Second-line combination antiretroviral therapy in resource-limited settings: facing the challenges through clinical research. AIDS.

[14] Galarraga O, O’Brien ME, Gutierrez JP, Renaud-Thery F, Nguimfack BD, Beusenberg M, Waldman K, Soni A, Bertozzi SM, Greener R (2007). Forecast of demand for antiretroviral drugs in low and middle-income countries: 2007-2008. AIDS.

[15] UNICEF WU: Global HIV/AIDS response: epidemic update and health sector progress towards universal access: progress report 2011. Available from http://www.who.int/hiv/pub/progressreports/en/index.html [Access date 1 March 2013]; 2013.,

[16] PAHO: Antiretroviral Treatment in the Spotlight: A Public Health Analysis in Latin America and the Caribbean. Available from http://www.paho.org/hq/index.php?option=com_docman&task=doc_view&gid=23710&Itemid [Access date 15 Jan 2014]; 2013.

[17] Grinsztejn B, Luz PM, Pacheco AG, Santos DV, Velasque L, Moreira RI, Guimaraes MR, Nunes EP, Lemos AS, Ribeiro SR, Campos DP, Vitoria MA, Veloso VG (2013). Changing mortality profile among HIV-infected patients in Rio de Janeiro, Brazil: shifting from AIDS to non-AIDS related conditions in the HAART era. PloS one.

[18] BRASIL: PROTOCOLO CLÍNICO E DIRETRIZES TERAPÊUTICAS PARA ADULTOS VIVENDO COM HIV/AIDS. Edited by Ministério da Saúde CNdDeA. http://www.aids.gov.br/pcdt/protocolo-clinico; 2013.

[19] Cardoso SW, Grinsztejn B, Velasque L, Veloso VG, Luz PM, Friedman RK, Morgado M, Ribeiro SR, Moreira RI, Keruly J, Moore RD (2010). Incidence of modifying or discontinuing first HAART regimen and its determinants in a cohort of HIV-infected patients from Rio de Janeiro, Brazil. AIDS Res Hum Retrovir.

[20] Napravnik S, Eron JJ, Sterling TR, Juday T, Uy J, Moore RD (2013). Outcomes of second combination antiretroviral therapy regimens among HIV-infected persons in clinical care: a multicenter cohort study. AIDS Res Hum Retrovir.

[21] von Wyl V, Klimkait T, Yerly S, Nicca D, Furrer H, Cavassini M, Calmy A, Bernasconi E, Boni J, Aubert V, Gunthard HF, Bucher HC, Glass TR, Swiss HIVCS (2013). Adherence as a predictor of the development of class-specific resistance mutations: the Swiss HIV Cohort Study. PloS One.

[22] Lima VD, Harrigan R, Bangsberg DR, Hogg RS, Gross R, Yip B, Montaner JS (2009). The combined effect of modern highly active antiretroviral therapy regimens and adherence on mortality over time. J Acquir Immune Defic Syndr.

[23] Torres TS, Cardoso SW, Velasque Lde S, Marins LM, Oliveira MS, Veloso VG, Grinsztejn B (2013). Aging with HIV: an overview of an urban cohort in Rio de Janeiro (Brazil) across decades of life. Braz J Infect Dis.

[24] Sigaloff KC, Hamers RL, Wallis CL, Kityo C, Siwale M, Ive P, Botes ME, Mandaliya K, Wellington M, Osibogun A, Stevens WS, van Vugt M, de Wit TF R, PharmAccess African Studies to Evaluate R (2012). Second-line antiretroviral treatment successfully resuppresses drug-resistant HIV-1 after first-line failure: prospective cohort in Sub-Saharan Africa. J Infect Dis.

[25] Waters L, Patterson B, Scourfield A, Hughes A, de Silva S, Gazzard B, Barton S, Asboe D, Pozniak A, Boffito M (2012). A dedicated clinic for HIV-positive individuals over 50 years of age: a multidisciplinary experience. Int J STD & AIDS.

[26] Silverberg MJ, Leyden W, Horberg MA, DeLorenze GN, Klein D, Quesenberry CP (2007). Older age and the response to and tolerability of antiretroviral therapy. Arch Intern Med.

[27] van Leth F, Andrews S, Grinsztejn B, Wilkins E, Lazanas MK, Lange JM, Montaner J, Group NNs (2005). The effect of baseline CD4 cell count and HIV-1 viral load on the efficacy and safety of nevirapine or efavirenz-based first-line HAART. Aids.

[28] Taiwo B, Zheng L, Gallien S, Matining RM, Kuritzkes DR, Wilson CC, Berzins BI, Acosta EP, Bastow B, Kim PS, Eron JJ, Team AA (2011). Efficacy of a nucleoside-sparing regimen of darunavir/ritonavir plus raltegravir in treatment-naive HIV-1-infected patients (ACTG A5262). Aids.

[29] Brinson C: Potential Implications of Baseline Viral Load on the Relative Potency of First-line, NNRTI-Based Antiretroviral Therapy. 2012, 1:542. doi:10.4172/scientificreports.542.

[30] Waters L, Bansi L, Asboe D, Pozniak A, Smith E, Orkin C, Fearnhill E, Dunn D, Phillips A, UK CHIC Study, UK HIV Drug Resustance Database: Second-line protease inhibitor-based antiretroviral therapy after non-nucleoside reverse transcriptase inhibitor failure: the effect of a nucleoside backbone. *Antivir Ther* 2013, 18(2):213–219., 10.3851/imp2329 23653911

[31] Koulla-Shiro S, Ciaffi L, Le Moing V, Ndour CT, Sawadogo A, Eymard-Duvernay S, Aghokeng A, Reynes J, Calmy A, Delaporte E, Group obotlS: Randomized Comparison of Three Second Line ART Regimens in Africa: the 2 Lady Study. In: *Conference on Retroviruses and Opportunistic Infections: 2014; Boston*; 2014. http://www.croiconference.org/sites/all/abstracts/541LB.pdf,

[32] Torres TS, Cardoso SW, Velasque LS, Veloso VG, Grinsztejn B (2014). Incidence rate of modifying or discontinuing first combined antiretroviral therapy regimen due to toxicity during the first year of treatment stratified by age. Braz J Infect Dis.

[33] Walmsley S, Avihingsanon A, Slim J, Ward DJ, Ruxrungtham K, Brunetta J, Bredeek UF, Jayaweera D, Guittari CJ, Larson P, Schutz M, Raffi F (2009). Gemini: a noninferiority study of saquinavir/ritonavir versus lopinavir/ritonavir as initial HIV-1 therapy in adults. J Acquir Immune Defic Syndr.

[34] Riddler SA, Haubrich R, DiRienzo AG, Peeples L, Powderly WG, Klingman KL, Garren KW, George T, Rooney JF, Brizz B, Lalloo UG, Murphy RL, Swindells S, Havlir D, Mellors JW, Team ACTGSA (2008). Class-sparing regimens for initial treatment of HIV-1 infection. New Eng J Med.

[35] Gupta RK, Goodall RL, Ranopa M, Kityo C, Munderi P, Lyagoba F, Mugarura L, Gilks CF, Kaleebu P, Pillay D, Group DV, Trial T (2014). High Rate of HIV Resuppression After Viral Failure on First-line Antiretroviral Therapy in the Absence of Switch to Second-line Therapy. Clin Infect Dis.

