# Supplementary figures and images for: Outcomes of second-line combination antiretroviral therapy for HIV-infected patients: a cohort study from Rio de Janeiro, Brazil
Source: BMC Infect Dis. 2014 Dec 19;14:699. doi: 10.1186/s12879-014-0699-5 (PMC4297410; doi:10.1186/s12879-014-0699-5)

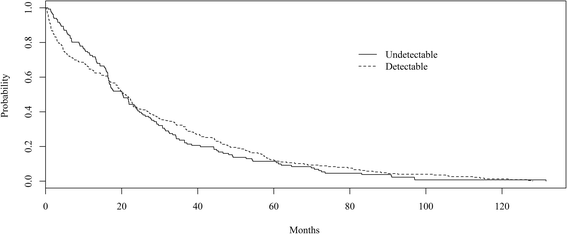

Supplement: Supplementary file 1 — Authors’ original file for figure 1 [file 12879_2014_699_MOESM1_ESM.gif]

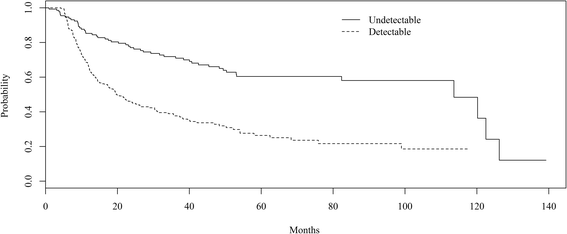

Supplement: Supplementary file 2 — Authors’ original file for figure 2 [file 12879_2014_699_MOESM2_ESM.gif]

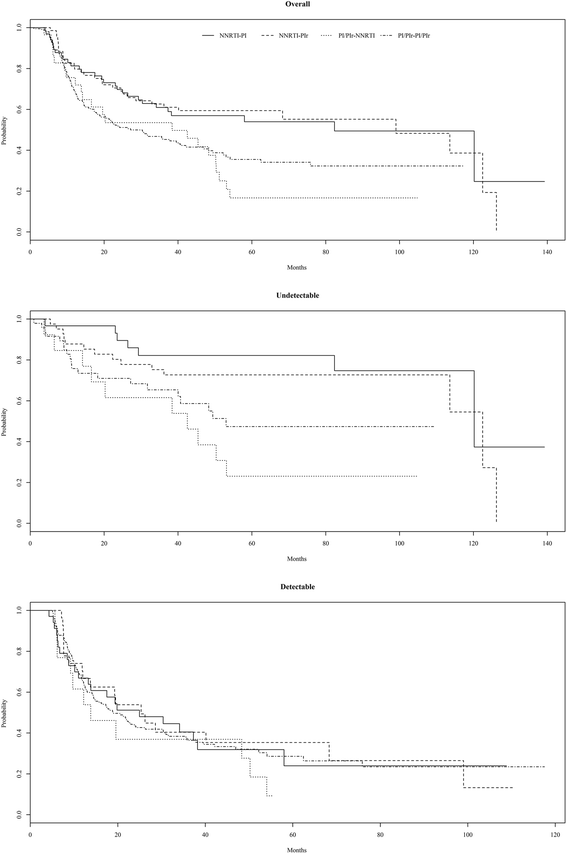

Supplement: Supplementary file 3 — Authors’ original file for figure 3 [file 12879_2014_699_MOESM3_ESM.gif]
